# Clinical features of pathological pseudoreligiosity in patients with mental disorders

**DOI:** 10.1192/j.eurpsy.2021.2041

**Published:** 2021-08-13

**Authors:** O. Borisova, G. Kopeyko, E. Gedevani, V. Kaleda

**Affiliations:** 1 Investigation Group Of Specific Psychopathological Forms At Department Of Youth Psychiatry, Federal State Budgetary Scientific Institution «Mental Health Research Center», Moscow, Russian Federation; 2 Department Of Youth Psychiatry, FSBSI «Mental Health Research Centre», Moscow, Russian Federation

**Keywords:** psychopathology, religious delusions, pathological pseudoreligiosity

## Abstract

**Introduction:**

The term pathological pseudoreligiosity (PPR) has been chosen for description of mental disorders with religious content (MDRC), accompanied with distortion of acceptance and assimilation of religious convictions, and with significant changes in patient’s religious behavior and way of life.

**Objectives:**

To assess the entire spectrum of mental pathology with religious content and relate it to the depth of mental disorder.

**Methods:**

857 patients (300 males, 557 females), with religious worldview and mental disorders were observed with psychopathological and follow-up methods.

**Results:**

The pathological pseudoreligiosity was detected in 326 patients – 38%. Follow-up period estimated mean 9,5 years. Next mental disorders with religious content were identified and described. Specific PPR types were correlated with register of the depth of mental disorder (K. Schneider):

**Table:** 

Types of PPR	Pts		The register of mental disorders
Toxic faith	6	1,8%	Personality disorders
Anorexia due to overvalued religious convictions	12	3,7%	Neurotic register
Depressive with congruent religious ideas of sinfulness, feeling of being abandoned by God	63	19,3%	Affective register
Depressive states with overvalued doubts of belief choice.	11	3,4%	
Overvalued religious behavior	13	4%	Affective-delusional
Delusion of spiritual hypochondria	7	2,2%	Delusional
Eschatological delusion	21	6,4%	
Anorexia in the form of delusional behavior with religious contents	11	3,4%	Hallucinatory-delusional
Apocalyptic delusion	32	9,8%	
Religious delusion	138	42,3%	
Religious standing, stiffening, mutism	4	1,2%	Catatonic
Fragmentary religious ideas	8	2,5%	Organic

**Conclusions:**

Management and treatment of patients suffering from MDRC with pathological pseudoreligiosity requires a particular approach. The consideration must be given to religious content of mental disorders and to clinical specifics of these disorders.

**Disclosure:**

No significant relationships.

